# Ethanol and Cognition: Indirect Effects, Neurotoxicity and Neuroprotection: A Review

**DOI:** 10.3390/ijerph7041540

**Published:** 2010-04-04

**Authors:** John C.M. Brust

**Affiliations:** Department of Neurology, Harlem Hospital Center and Columbia University College of Physicians & Surgeons, New York, NY 10037, USA; E-Mail: jcb2@columbia.edu

**Keywords:** Wernicke-Korsakoff, alcoholic dementia, glutamate

## Abstract

Ethanol affects cognition in a number of ways. Indirect effects include intoxication, withdrawal, brain trauma, central nervous system infection, hypoglycemia, hepatic failure, and Marchiafava-Bignami disease. Nutritional deficiency can cause pellagra and Wernicke-Korsakoff disorder. Additionally, ethanol is a direct neurotoxin and in sufficient dosage can cause lasting dementia. However, ethanol also has neuroprotectant properties and in low-to-moderate dosage reduces the risk of dementia, including Alzheimer type. In fetuses ethanol is teratogenic, and whether there exists a safe dose during pregnancy is uncertain and controversial.

## 1. Introduction

The term “alcoholic” is applied to those who are psychically dependent (addicted) to ethanol—that is, craving for ethanol is a daily preoccupation. It is also applied to those who are physically dependent on ethanol—that is, cessation of drinking causes physical withdrawal symptoms and signs. The term is sometimes extended to include “problem drinkers”, who may be neither psychically nor physically dependent on ethanol but who get into trouble when they drink [1]. In the United States it is estimated that 7% of adults and 19% of adolescents are alcoholics or problem drinkers and that ethanol accounts for more than 100,000 deaths per year or 5% of all mortality [2]. As with other reinforcing drugs, alcohol addiction—*i.e.*, compulsive use, craving—depends on mesolimbic dopaminergic pathways of the so-called “reward system” [3].

## 2. Indirect Effects

Ethanol can alter mentation in a variety of ways. Affecting many neurotransmitter systems, ethanol inhibits excitatory glutamate receptors and facilitates inhibitory γ-aminobutyric acid (GABA) receptors [4]. Early symptoms of acute intoxication—euphoria and disinhibition—progress to stupor and respiratory depression [5]. Perhaps reflecting glutamate receptor up-regulation and GABA-receptor down-regulation, abrupt abstinence after prolonged or binge drinking can result in tremor, hallucinations (visual, auditory, or tactile), seizures, or delirium tremens, with severely constricted attentiveness, fluctuating levels of alertness, agitation, and autonomic instability [6–8]. It is possible, moreover, that repeated binges and withdrawals cause not only early abstinence symptoms but also glutamate-induced excitotoxicity and permanent neuronal damage, in turn contributing to more lasting neurological disorders, including dementia.

Other causes of acutely altered mentation in heavy drinkers include cerebral trauma [9], meningitis [10], hypoglycemia [11], hepatic encephalopathy [12], alcoholic ketoacidosis [13] and concomitant use of other agents such as cocaine or heroin [1]. Marchiafava-Bignami disease, a rare disorder nearly always diagnosed in alcoholics, causes mania, depression, paranoia, and dementia, plus seizures, paresis, and ataxia, and often progresses to coma and death within a few months; symptoms are not readily explained by the prominent corpus callosum demyelination that is the pathological hallmark of this poorly understood disease [14].

## 3. Nutritional Deficiency Disorders

Often deficient in thiamine, nicotinic acid, other B vitamins, and folate, alcoholics frequently develop neurological disorders associated with malnutrition, including cerebellar degeneration, amblyopia, polyneuropathy, and disorders affecting cognition. In pellagra nicotinic acid deficiency results in skin, gastrointestinal, and mental abnormalities which can progress to memory impairment, delusions, hallucinations, dementia, or delirium; hypertonus and startle myoclonus may be present. Symptoms usually improve following treatment with nicotinic acid or nicotinamide [15].

A more frequently encountered nutritional disorder in alcoholics is Wernicke–Korsakoff disease [16]. Wernicke and Korsakoff syndromes share the same pathology—characteristic histological abnormalities within the medial and anterior thalamus, hypothalamus (including the mammillary bodies), and periaqueductal grey matter of the midbrain, but they have different clinical features. Wernicke syndrome consists of abnormal mentation, abnormal eye movements, and gait ataxia. Mental abnormalities include varying combinations of inattentiveness, abulia (apathy), and impaired memory progressing in the absence of treatment to coma. Eye movement abnormalities begin with limitations of abduction or horizontal gaze and progress to ophthalmoplegia. Gait ataxia progresses to inability to stand. Patients are thiamine-deficient, and symptoms rapidly improve when thiamine is replaced in a timely fashion. Improvement is often less than complete, however. A patient may be left with nystagmus and a broad-based gait, and the multi-domain cognitive impairment may evolve into a more selective amnestic disorder—Korsakoff syndrome. How often Korsakoff syndrome occurs in the absence of antecedent Wernicke syndrome is uncertain. Autopsies have revealed Wernicke-Korsakoff pathology in patients unsuspected of having Wernicke syndrome prior to death [17].

Clinical evidence suggests that Wernicke syndrome is more likely to occur in nutritionally deficient alcoholics than in comparably deficient non-alcoholics. In developing countries, thiamine deficiency in a non-alcoholic is more likely to produce beri-beri with cardiac failure and polyneuropathy than Wernicke syndrome [18]. (On the other hand, several of Korsakoff’s original patients had not been heavy drinkers [19].) A biologically plausible mechanism by which thiamine deficiency and ethanol could have additive or even synergistic effects on cognition involves glutamate. Thiamine deficiency causes excessive glutamate release and thus like ethanol has the potential to cause excitotoxic neuronal damage [20]. When thiamine-induced glutamate release is combined with ethanol-induced glutamate receptor up-regulation, the potential for excitotoxicity would be compounded. In anecdotal reports patients with Wernicke-Korsakoff syndrome have experienced symptomatic improvement following treatment with the glutamate NMDA receptor antagonist memantine [21,22]. (They have also reportedly improved after treatment with cholinergic agents such as donepezil and rivastigmine [23,24].)

A combined effect of nutritional deficiency and direct ethanol toxicity appears operative in other neurological disorders encountered in alcoholics. Cerebellar degeneration can occur with severe nutritional deprivation in both drinkers and non-drinkers [25,26]. Animal studies, however, demonstrate neurotoxic effects of ethanol on cerebellar granule and Purkinje cells [27,28]. Optic atrophy in heavy drinkers (formerly called “tobacco-alcohol amblyopia”) improves with nutritional supplementation, but it is likely that ethanol toxicity (as well as, perhaps, neurotoxic compounds in tobacco smoke) are contributory [29]. Polyneuropathy in alcoholics similarly appears to have two causes. In a study comparing polyneuropathy in thiamine-deficient drinkers, thiamine-deficient non-drinkers, and alcoholics without thiamine deficiency, polyneuropathy attributable to thiamine deficiency alone was motor-dominant and rapidly progressive, impaired both superficial and deep sensation, and caused predominantly large-fiber axonal loss. Polyneuropathy in alcoholics without thiamine deficiency was sensory-dominant, slowly progressive, impaired superficial sensation, and caused predominantly small-fiber axonal loss. Thiamine-deficient alcoholics tended to have a mixture of the two types [30].

## 4. Alcoholic Dementia

In recent decades it has become increasingly evident that ethanol can cause lasting cognitive impairment—“alcoholic dementia” or “ethanol-related dementia”—in the absence of nutritional deficiency, cerebral trauma, hepatic failure, or other indirect forms of brain injury. Many alcoholics demonstrate gradually progressive multi-domain cognitive impairment rather than a more restrictive amnestic disorder, a prior history of Wernicke syndrome is often lacking, and there may be little evidence of past or present nutritional deficiency. Computerized tomographic (CT) scanning shows ventricular and sulcal enlargement unexplained by the neuropathology of Wernicke-Korsakoff syndrome, and the observed brain shrinkage reportedly improves with abstinence [31–33].

Ethanol-related dementia has been estimated to represent roughly 10% of all cases of dementia, and “heavy alcohol use” probably contributes to many more [32,33]. Proposed criteria for ethanol-related dementia include dementia for at least 60 days after last exposure to ethanol, minimum 35 standard drinks per week for males and 28 for females for more than 5 years, and significant ethanol use within 3 years of the onset of impaired cognition [34,35].

Animal studies using pair-fed controls confirm that ethanol is neurotoxic, and in exposed animals neuropathological changes correlate with impaired memory and learning [36,37]. Reported abnormalities include loss of hippocampal CA1 and CA3 pyramidal neurons, mossy fiber-CA3 synapses, and dentate granule cells; loss of cholinergic neurons in the basal forebrain; pathological changes in neurons of cerebral cortex, hypothalamus, and brainstem; and impaired pruning of redundant cortical synapses during early development [38–44]. Damage is dose-related and especially likely with binge drinking that produces high blood ethanol concentrations (BEC) [45].

Nutritional deficiency can be difficult to exclude in cognitively impaired heavy drinkers, but there is convincing evidence of brain damage in “uncomplicated alcoholics” [46]. Transcranial magnetic stimulation in chronic alcoholics demonstrates significant prolongation of central motor conduction time [47]. Quantitative neuroimaging suggests association of cognitive impairment with damaged pontocerebellar and cerebellothalamocortical systems [48]. Cerebral white matter volume loss is prominent, and MRI, including diffusion-weighted imaging, reveals involvement of structural elements rather than water loss [49,50]. Such changes in cerebral white matter are at least partly reversible, although it is less clear whether cognitive improvement accompanies improvement on imaging [51,52]. Magnetic resonance spectroscopy reveals damage to white matter phospholipids in the absence of white matter volume loss [53]. White matter abnormalities, including excessive intra- and extracellular fluid, have also been identified with diffusion tensor imaging [54].

In human studies neuronal loss is described in many brain areas, most consistently the superior frontal association cortex, hypothalamus, and cerebellum, and less consistently the hippocampus, amygdala, and locus coeruleus [46]. Basal ganglia and serotonergic raphe nuclei appear to be unaffected [32,55]. Using the neuronal/axonal marker N-acetyl aspartate (NAA), magnetic resonance spectroscopy confirmed the special vulnerability of pre-frontal cortex in alcoholics, and abnormalities in planning, organization, problem solving, and abstracting, as well as lack of insight, disinhibition, and perseveration are consistent with these regional vulnerabilities [55–58]. Early changes in dendrites, receptors, and neurotransmitters probably produce cognitive impairment in advance of gross morphological change [59]. Women appear to be more susceptible than men to the adverse effects of ethanol, exhibiting earlier changes yet greater recovery with abstinence [60]. The likelihood of cognitive impairment may be greater in subjects with repeated prior episodes of symptomatic withdrawal [61].

Ethanol neurotoxicity may have several mechanisms, including glutamate excitotoxicity and oxidative stress, exacerbated in some cases by thiamine deficiency [55,62–64]. In an open label study of 19 patients with probable ethanol-related dementia, cognitive improvement followed treatment with the glutamate NMDA receptor antagonist memantine [21]. (As noted, Wernicke-Korsakoff syndrome also reportedly benefits from memantine therapy [22].) In rat hippocampal slice cultures subjected to 10 days of in vitro ethanol followed by 24 hours of withdrawal, memantine effectively blocked ethanol withdrawal-induced neurotoxicity [65]. (Interestingly, memantine also appears to reduce craving in subjects dependent on ethanol [66].)

Homocysteine is also implicated in ethanol neurotoxicity; hyperhomocysteinemia follows folate deficiency, and homocysteine acts as an agonist at glutamate NMDA receptors, increasing NMDA receptor transmission and the potential for excitotoxicity [67–69]. Ethanol also reduces the availability of brain-neurotrophic factor and nerve growth factor, possibly resulting in impaired intracellular signaling pathways [70]. Ethanol-induced DNA strand breaks might cause neuronal death [71]. In animals protein adduct formation with the ethanol metabolite acetaldehyde was found in frontal lobe cortex and white matter [72].

Just as thiamine deficiency may interact synergistically with alcohol neurotoxicity to produce cognitive impairment, other neurological disorders encountered in alcoholics, including hepatic encephalopathy and prior cerebral trauma or hypoglycemia, could also be contributory. Also operative might be age of onset of drinking and genetic vulnerability [73,74].

Attempts to define a safe dose threshold for ethanol have been inconsistent. A review of 19 published studies addressing this issue concluded that 5 or 6 “standard drinks” per day over extended periods resulted in “cognitive inefficiencies”, that 7 to 9 drinks per day resulted in “mild cognitive deficits”, and that 10 or more drinks per day caused impaired cognition of a degree encountered in frank alcoholics [75]. In some studies heavy ethanol consumption (average 418g ethanol per week) correlated with reduced frontal lobe volume, whereas moderate consumption (181g per week) and light consumption (average 88g per week) did not [76]. Some studies, moreover, found that light-to-moderate ethanol intake *reduced* the likelihood of dementia [77–86].

## 5. Ethanol as a Neuroprotectant

### 5.1. Reducing the Risk of Coronary Artery Disease and Ischemic Stroke

Relevant to such a protective effect of mild-to-moderate drinking on cognitive decline are its complex effects on coronary artery disease (CAD) and ischemic stroke. Numerous epidemiological studies, including prospective cohort studies, provide powerful evidence that one half to 2 drinks per day reduce the risk of myocardial infarction by roughly 25%, with 6 or more drinks per day increasing the risk [87–89]. The result is a J-shaped curve, with mild-to-moderate ethanol intake reducing risk of CAD compared to non-drinking but heavy ethanol intake increasing risk. Concern has been expressed that the apparent protective effect is overestimated when non-drinkers are combined with former drinkers, who might have stopped drinking for reasons of ill health [90,91]. However, separating former drinkers from longer-term abstainers in a large cohort study did not alter the results [92,93]. Similarly, the protective effects remained after correcting for sociodemographic and other clinical characteristics [93]. Benefit has been found for red and white wine, beer, and liquor [93].

A number of mechanisms appear to explain ethanol’s protective effect. Ethanol raises blood levels of high density lipoprotein cholesterol (HDL-C) in a dose-dependent fashion, and some studies suggest that this effect accounts for at least half of the protection against CAD [94]. Ethanol also lowers high-density lipoprotein cholesterol, increases insulin sensitivity [95], prevents platelet aggregation [96], increases fibrinolysis [97], opposes thrombin activity [87], and reduces inflammatory markers [98]. In addition, animal studies demonstrate a direct protective effect of ethanol on cardiac myocytes rendered ischemic; this effect has been linked to ethanol’s interactions with protein kinase C, adenosine receptors, and “cardioprotective proteins” that include superoxide dismutase, nitric oxide synthase, and heat shock proteins [87]. It is speculated that some of the benefit might be attributable to antioxidant polyphenols such as resveratrol, which are especially abundant in red wine [99–101]. In a rat model of cerebral infarction resveratrol reduced infarct volume, and the neuroprotection correlated with downregulation of inducible nitric oxide synthase (iNOS) and upregulation of endothelial nitric oxide synthase (eNOS) [102].

A similar J-shaped curve describes the association of ethanol intake and ischemic stroke, although the protective effects of low-to-moderate intake are less dramatic. Meta-analysis of 19 cohort studies and 16 case-control studies (selected from a total of 122 reports) found that compared with abstention, consumption of less than 12 g ethanol per day reduced the risk of total stroke (relative risk, RR: 0.80); consumption of 12 to 24 g per day reduced the risk of ischemic stroke (RR: 0.72); and consumption of more than 60 g per day increased the risk of total stroke (RR: 1.64), ischemic stroke (RR:1.69), and hemorrhagic stroke (RR: 2.18). Light-to-moderate ethanol intake did not reduce the risk of hemorrhagic stroke [103]. Subsequent cohort studies have shown comparable results [104,105]. It is likely that similar effects on serum lipoproteins, coagulation factors, and platelets contribute to the reduced risk of both myocardial infarction and ischemic stroke conferred by low doses of ethanol, and antioxidants such as resveratrol (as well as the antioxidant properties of ethanol itself) might provide neuroprotection to the brain [87]. In animal models of ischemic stroke, prior ethanol reduced delayed neuronal death, neuronal and dendritic degeneration, oxidative DNA damage, glial cell activation, and neutrophil infiltration [106].

### 5.2. Reducing the Risk of Dementia

The possibility that ethanol confers direct neuroprotection to the brain has obvious bearing on the apparent risk reduction of dementia in selected drinkers. Once again a J-shaped curve emerges, with moderate intake reducing the risk of cognitive impairment and heavy drinking increasing it [77,78]. In the French PAQUID Study dementia was less prevalent among wine-drinkers, implicating anti-oxidant polyphenols, but few subjects in that study drank beer or liquor [79,107,108]. Studies from around the world include the French PAQUID Study [79,107,108], the French Epidemiology of Vascular Aging Study [109], the National Heart Lung and Blood Institute Twin Study [110], the Rotterdam Study [80], the Copenhagan City Heart Study [81], the Chinese Nanjing University Study [111], the Italian Longitudinal Study on Aging [112,113], the Cardiovascular Health Study [82], the Nurses’ Health Study [114], the Washington Heights Inwood Columbia Aging Project [115], and the Prospective Population Study of Women in Göteborg, Sweden [116].

Studies have been both cohort [80,109–116] and case-control [81,82], and subjects’ ages at enrollment have varied—in one study 59 to 69 years [110], in another 70 to 81 years [114]. Study design has included enrollment of subjects considered cognitively normal or “non-demented” at outset [80,81], enrollment of subjects with “mild cognitive impairment” [111,112], and case-control analysis of demented subjects [82]. In some studies protection from cognitive decline was conferred only by wine [79,81,107,108,115,116]; others found either wine, beer, or liquor to be protective [80,82,112,114]. In some studies, benefit was evident only in subjects carrying an APOEɛ4 allele, or an APOEɛ4 allele enhanced the protective effect [80,110]. In other studies, benefit was evident only in subjects lacking an APOEɛ4 allele, or an APOEɛ4 allele reduced the protective effect [82,109,112,115]. One study found no effect of APOEɛ4 genotype on risk reduction [114].

A 2008 meta-analysis reviewed 23 studies addressing the association of ethanol and incident dementia or cognitive decline [117]. Twenty studies were epidemiological cohort and 3 were case-control nested in a cohort. The authors concluded that small amounts of ethanol probably protect against dementia (RR: 0.63) and Alzheimer disease (RR: 0.57) but not against vascular dementia (RR: 0.82, non-significant) or cognitive decline (RR: 0.89, non-significant). Studies varied as to what constituted optimal consumption or what defined a “standard drink.” Overall the evidence supported special benefit conferred by wine (but little evidence to favor red wine over white wine) and greater benefit in subjects lacking an APOEɛ4 allele. Two studies addressing concern that combining former drinkers with non-drinkers might result in spurious protection found that the positive benefit for ethanol was maintained when former drinkers were excluded [82]. The authors of the meta-analysis emphasized that association is not the same as causation and that moderate drinkers, compared to abstainers and heavy drinkers, might “live healthier lives”.

Mild-to-moderate consumption of ethanol nonetheless appears to reduce the risk of dementia among older people, and the favorable effects of ethanol on cerebrovascular disease do not explain the benefit in non-vascular dementia. Animal models are consistent with these observations. “Alcohol-preferring” rats chronically consuming 15% ethanol/water were protected from apoptosis caused by inflammatory lipopolysaccharide injection [119]. In brain cultures non-neurotoxic ethanol exposure protects against excitotoxic NMDA receptor mediated neurodegeneration, and the benefit paralleled induction of heat shock proteins [120,121]. Neuroprotective effects, including those of polyphenol antioxidants as well as the antioxidant effects of ethanol itself, are plausible mechanisms for these effects. The Rotterdam Study investigators speculated that the greater benefit of ethanol among subjects with an APOEɛ4 allele might be related to ethanol’s ability to block oxidation of the apolipoprotein, thereby preventing it from binding to β-amyloid [80]. Most studies, however, showed greatest benefit among subjects lacking an APOEɛ4 allele [117].

Perhaps surprisingly, the apparent neuroprotection observed in most epidemiological studies has not been reflected in imaging studies. Framingham Study investigators tested the hypothesis that moderate ethanol consumption would be associated with less age-related brain volume reduction and white matter lesions compared to either no drinking or heavy drinking [122]. Not only was moderate ethanol intake not protective, but reduction in brain volume had a negative linear association with the amount of ethanol consumed (“abstainers”, “former drinkers”, “low”, “moderate”, “high”). Other studies also reported increased ventricular size [123,124], reduced grey matter volumes [125], or “brain atrophy” [126] with increasing amounts of ethanol consumption. Two studies did demonstrate fewer cerebral white matter lesions in moderate drinkers [123,127].

## 6. Ethanol’s Effects on the Fetus

The fetal effects of ethanol involve a different kind of neurotoxicity. The fetal alcohol syndrome (FAS) is a triad consisting of CNS dysfunction, intrauterine growth deficiency, and distinctive facial dysmorphism; less often there are anomalies of the heart, skeleton, urogenital organs, skin and muscles [128,129]. Symptoms include mental retardation, hypotonia, poor coordination, hyperactivity, and behavioral problems. Neuropathological features include microcephaly, abnormal cortical thickness, reduced cerebral white matter volume, and abnormalities of the corpus callosum and cerebellar vermis. Long-term follow-up demonstrates that mental retardation, abnormal behavior, and facial dysmorphism persist into adulthood [130]. It is also evident that cognitive and behavioral abnormalities can occur in the absence of dysmorphisms, so-called “fetal alcohol effects”, “alcohol-related neurodevelopmental disorder”, (ARND) or “fetal alcohol spectrum disorders” [131–136].

Carefully controlled animal studies confirm that ethanol toxicity is the cause of FAS and that lower doses can produce impaired mental ability without other physical signs [137]. Interestingly, although in humans with FAS the hippocampi are reportedly normal-sized, in rodents exposed in utero to ethanol the hippocampi display reduced number of neurons and dendritic spine density, correlating with the animals’ impaired learning and memory [138]. Ethanol disrupts numerous developmental events in animal models, including neurogenesis, cell migration, cell adhesion, neuron survival, axon outgrowth, synapse formation, and neurotransmitter function [139–141]. Animal studies include reports that “low-to-moderate” amounts of ethanol, a binge pattern of consumption, or even single exposures, can cause features of FAS [142–144].

Epidemiological studies in humans similarly raise the question of whether a “safe dose” of intrauterine ethanol exposure exists, with reports of FAS, low birth weight, decreased head circumference, dysmorphism, or subtle neurological and behavioral effects associated with 100 g ethanol per week, 10 g per day, or even 0.1 oz. per day [145–148]. The validity of animal and human studies claiming teratogenicity of such low doses has not gone unchallenged, however, and whether there is a threshold of safety remains controversial [149–150]. So are estimates of FAS prevalence, which range from 0.5 to 2.0 per 1000 births in the United States [151] to 0.06 per 1000 births in Australia [152] to 3.7–7.4 per 1000 in the Lazio province of Italy [153].

Proposed mechanisms for ethanol teratogenicity (not mutually exclusive) include vasospasm and CNS ischemia [154]; blockade at glutamate NMDA receptors (which in fetal brain play a crucial role in neuronal differentiation) [155]; inhibition of the action of a neuronal cell adhesion molecule, L1, which in fetal brain mediates neurite outgrowth [156]; and excessive activation of glycogen synthase kinase 3β, a serine/threonine kinase that regulates fetal neurogenesis, neuronal migration, synapse formation, and neuronal survival [141].

## 7. Conclusions

Identifying the mechanisms of ethanol’s multiple effects on cognition—including neurotoxicity, neuroprotection, interaction with nutritional deficiency, and teratology—will have obvious bearing not only on medical management but also on public policy.
